# Application of Atmospheric-Pressure Plasma Treatment in Anti-Hairfalling of Polyester–Cotton Fleece Knitted Fabrics

**DOI:** 10.3390/polym15092097

**Published:** 2023-04-28

**Authors:** Zhipeng Chen, Zhong Zhao, Jihong Wu

**Affiliations:** State Key Laboratory of New Textile Materials and Advanced Processing Technologies, Wuhan Textile University, Wuhan 430073, China

**Keywords:** APP treatment, polyester–cotton fleece knitted fabrics, anti-hairfalling properties

## Abstract

In this study, atmospheric-pressure plasma (APP) was used to modify the surface of polyester–cotton fleece knitted fabrics to improve their anti-hairfalling properties. A series of treated samples were obtained by changing the power of plasma and treatment time. Scanning electron microscopy (SEM) and the surface roughness results reveal that the APP treatment can increase the roughness of fibers. The withdraw force and hairiness length of fibers results indicate that increasing withdraw force and decreasing hairiness length of fabrics can reduce hairfalling of the fibers. The values of weight loss rate confirm APP-treated powers and times can influence anti-hairfalling properties of fabrics. In addition, the best APP-treated time and electric power for the anti-hairfalling properties of the treated fabrics are respectively 15 s and 1.0 kW. Under this condition, the anti-hairfalling properties of the treated fabrics are improved by 48.3%, the contact angle decreased by 39.7%, and the wicking height increased by 18.3% compared with the untreated fabrics. It is notable that APP treatment does not affect the handle and tensile properties of fabrics.

## 1. Introduction

The plasma surface treatment of fabrics is a dry technique that has the advantages of being an energy-saving, clean and sustainable method [1,2,3,4,5,6]. It has been increasingly used in many industrial applications such as material processing, pollution control, sterilization of medical instruments, plasma display panels, Ozone production, etc. [7,8,9,10,11]. In addition, plasma is the fourth state of matter, which is a gas composed of ions, free electrons, photons, neutral atoms, and molecules in the ground and excited states [12,13,14,15]. Studies have shown that plasma technology has been considered as an i excellent method of surface modification because it can functionalize the surface of the material while maintaining its original physical properties [16]. Plasma treatment has been used to modify the surface of various fabric materials and fibers [17,18,19,20,21,22], including the polymer substrate [23,24,25,26,27]. Among these applications, plasma can be also used for anti-hairfalling finishing of fabrics [28,29,30,31]. These studies show that the reactive species in the plasma can interact with the surface of the materials and modify some physical and chemical characteristics such as roughness, oxidation, etching, etc. [32,33,34].

Fleece knitted fabrics have relatively high mass and thickness and are widely used as an outdoor garment for activities and sportswear [35]. However, the twist of the fleece yarn on the surface is low which leads to the low cohesion of the fibers, thus reducing the withdraw force between the fibers in the yarn. The low withdraw force and long hairiness of fiber can lead to hairfalling. Therefore, it is necessary to improve the anti-hairfalling properties of polyester–cotton fleece knitted fabrics. Notably, the surface of polyester fiber is smooth without hairiness, thus improving the withdraw force between the cotton fibers and hairiness of fiber and reducing hairiness of fibers are the key factors in solving the problem of hairfalling. Nevertheless, there are few works of literature regarding the influence of APP on the anti-hairfalling of polyester–cotton fleece knitted fabrics. 

In this study, polyester–cotton fleece knitted fabrics was subjected to APP surface modification to improve anti-hairfalling property of the fabrics. We have investigated the morphological changes and withdraw force of hairiness of fibers that take place on the surface of polyester–cotton fleece knitted fabrics using SEM (scanning electron microscope), Wool HandleMeter, magnifier, millimeter-scale and Electronic Fabric Strength Tester when the fabric is exposed to APP. In addition, the anti-hairfalling properties changes at the surface of the fabric are further confirmed by an intensive study of the effect of various APP treatment parametric, and thereby determine the optimal APP anti-hairfalling treatment process. In addition, the wettability, handle, and mechanical properties of the treated fabrics are characterized by the contact angle, wicking height, handle, and tensile properties.

## 2. Experimental

### 2.1. Materials

The samples of polyester–cotton fleece knitted fabrics were provided by Taiju Textile Co., Ltd., Shanghai, China. The yarn of fleece knitted fabrics does not have sizing agent added in the manufacture of the fabric. The process parameters of the fabrics are as follows: the face yarn count is 19 tex, which is made of 50% cotton, 25% recycled polyester, and 25% viscose; the ground yarn count is 8 tex, which is made of 100% recycled polyester; the fleece yarn count is 49 tex, which is made of 70% cotton and 30% recycled polyester. In addition, the weight of the fabric is 300 g/m^2^. The deionized water used in this study is purified by the reverse osmosis water treatment system.

### 2.2. APP Treatment

**Figure 1:**
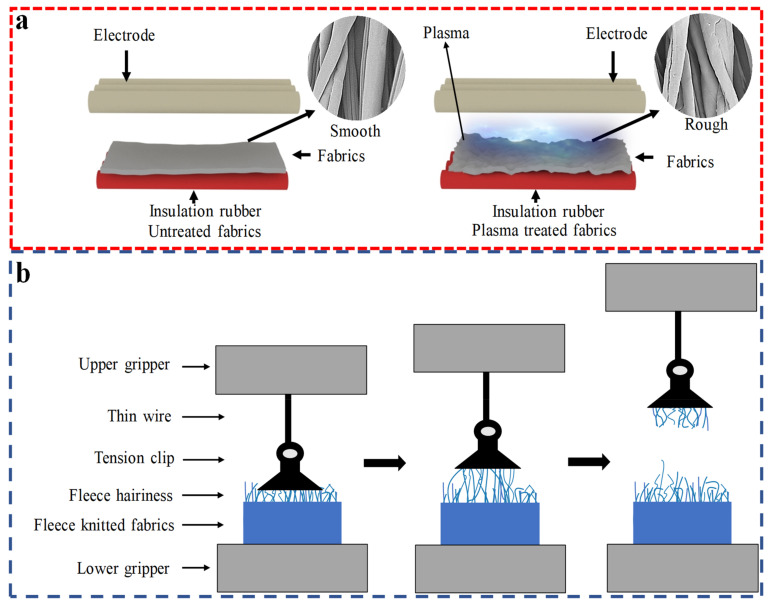
(**a**) Schematic diagram of the APP treatment technology; (**b**) Schematic diagram of the withdraw force of hairiness test.

Firstly, the fabrics were placed in the dark at 20 ± 2 °C temperature and 65 ± 5% humidity for 24 h before APP treatment. Secondly, the treatment was carried out in PG-10000F APP equipment (Suman Co., Ltd., Nanjing, China). The device consists of two electrodes, two rubber rollers, an air compressor, and a control host. In addition, atmospheric gas was the working gas and the working power range of the device was 0–9.9 kW, the jet distance was 20 mm, the electrode length was 1550 mm, and the moving speed of fabrics were 5.5 cm/s. The fleece surfaces of polyester–cotton fleece knitted fabrics were modified under the conditions of APP treatment with electric power of 1.0 kW at treatment time of 15–90 s and of APP treatment with treatment time of 15 s at electric power of 1.0–4.0 kW. The optimized APP condition was determined by the weight loss rate of polyester–cotton fleece knitted fabrics experiments. As shown in Figure 1a, the APP treatment was carried out in a conditioned environment (temperature 20 ± 2 °C and relative humidity of 65 ± 5%).

### 2.3. Morphologies

The surface morphology of the fleece surface of the polyester–cotton fleece knitted fabrics was scanned by scanning electron microscopy (SEM, Phenom ProX, Phenom Scientific Instruments Co., Ltd., Shanghai, China) before and after the treatments. A Wool HandleMeter (AWTA Co., Ltd., Kensington, Australia) was used to evaluate the handle and roughness of the samples. The index of roughness was chosen as the main parameter to evaluate the change of morphology caused by the APP treatment. These fabrics were cut into three samples in the same circular shape with a diameter of 113 mm and then put into the test chamber, and tested following the standard specified by Zhao et al. [36]. Average values for the handle measure values were then obtained from these three measurements. The hairiness length of the samples was characterized by a magnifier and millimeter scale. The fabrics were cut into 60 mm × 60 mm samples and then half was brushed in the transverse direction with the hairiness facing outwards. We used a magnifying glass to locate the hairiness and a millimeter scale to measure the length of the hairiness. The measurement was repeated ten times at different positions of the samples. Average values for the hairiness length of fleece surface were then obtained from these ten measurements. The withdraw force of the hairiness of the samples was characterized by the YG065H series Electronic Fabric Strength Tester (Laizhou Electronics Co., Ltd., Laizhou, China). The fabrics were cut into 60 mm × 60 mm samples and then fled in half in the transverse direction with the hairiness facing outwards. The upper gripper of the YG065H series Electronic Fabric Strength Tester held a thin wire with a tension clip at one end. The tension clip gripped the plush hairiness, and the lower gripper gripped the fabrics, as shown in Figure 1b. The distance between the upper and lower grippers was 80 mm and the stretching speed was set as 40 mm/s. The measurement was repeated ten times at different positions of the samples. The maximum value recorded during the test was set as the withdraw force value. Average values for the withdraw force were then obtained from these ten measurements.

### 2.4. Anti-Hairfalling Property

The samples treated by APP were subsequently subjected to weight loss tests to further evaluate the anti-hairfalling property of the polyester–cotton fleece knitted fabrics. The three individual samples were put into the YG502 series Fabric Fuzzing and Pilling Instrument (Laizhou Electronics Co., Ltd., Laizhou, China) for 500 friction tests, in which the test pressure was 790 cN. After the friction process, the samples were weighed, and the weight loss rate of the samples were calculated according to Equation (1). Normally, a larger value of the weight loss rate of the fabrics indicates worse anti-hairfalling property of the fabrics.

(1) $$P=\frac{M_{2}{-M}_{1}}{M_{0}}\times 100\%$$

where, P, M_0_, M_1_ and M_2_ are the weight loss rate of fabric in percentage, effective constant weight for fabric test in g, fabric weight before friction test in g, and fabric weight after friction test in g, respectively.

### 2.5. Wettability

The wettability of the treated samples was evaluated. The contact angle and wicking property were the two parameters employed to analyze the wettability of fabrics obtained under optimal treating conditions before and after APP treatment. Contact angle measurements were performed using the PGX Goniometer (Sindin Precision Instrument Co., Ltd., Dongguan, China) to test following the DB44/T1872-2016 Standard. The measurement was repeated ten times at different positions of the samples. Average values for the contact angles were then obtained from these ten measurements. The wicking property measurement were performed using the YG(B)871 series Wicking Effect Tester (Fangyuan Instrument Co., Ltd., Wenzhou, China). The fabrics were cut into three 200 mm × 20 mm samples and tested following the FZ/T01071-2008 Standard. The average capillary height within the range of 1 and 30 min were then obtained from these three measurements.

### 2.6. Tensile Test

By testing the tensile properties of the fabrics obtained under the optimal anti-hairfalling treatment conditions before and after APP treatment, the effects of APP treatment on the mechanical properties of the fabrics were analyzed. Tensile property measurements were performed using the YG065H series Electronic Fabric Strength Tester. The fabrics were cut into 300 mm × 50 mm samples and tested by a YG065H series Electronic Fabric Strength Tester following the GB/T3923.1-2013 Standard. The average values of breaking strength and elongation measurements were then obtained from these five measurements.

## 3. Results and Discussion

### 3.1. Surface Characteristic

#### 3.1.1. Morphology

**Figure 2:**
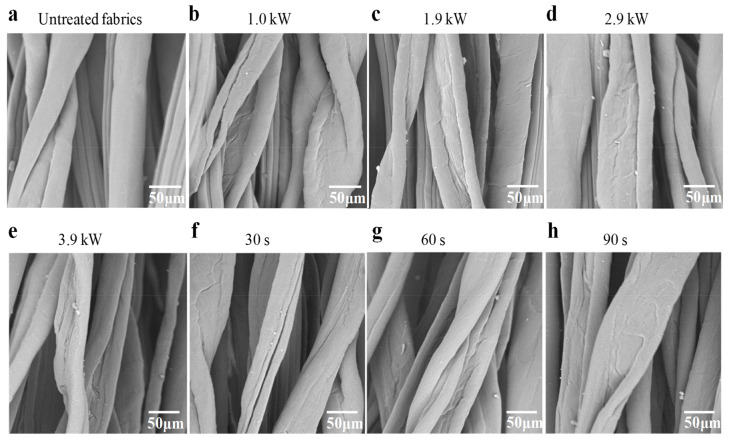
The SEM images of the samples treated under different conditions.

The SEM of untreated polyester–cotton fleece knitted fabrics are illustrated in Figure 2a. Smooth fiber surfaces can be observed. As the SEM images of the APP-treated fibers (displayed in Figure 2b–h at different powers and times) show, cracks and pits emerged on the fiber surface after the APP treatment. At higher power (3.9 Kw) and long time (90 s) the surface pattern surface pattern became more prominent. The reason is that the high-energy plasma hits the surface of the fiber, so that the surface obtains greater kinetic energy, and the impurities on the surface of cotton fiber are degraded and volatilized. At the same time, the continuous covering state of wax on the surface of cotton fiber is oxidized and destroyed, so that the fiber surface is etched. In addition, surface failure phenomena such as pits and cracks appear on the surface of fabric fibers, indicating that in addition to etching, plasma will also cause crack on the fiber surface, or bombard the fiber surface to fragment.

#### 3.1.2. Surface Roughness

**Figure 3:**
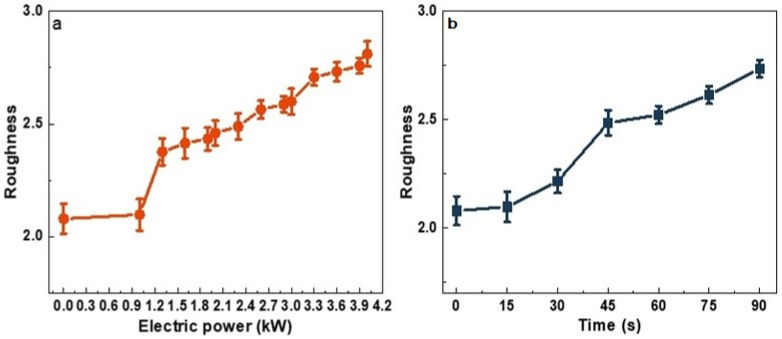
(**a**) Effect of APP treatment power on roughness of fabrics; (**b**) effect of APP treatment time on roughness of fabrics.

The roughness indexes of untreated and APP treated polyester–cotton fleece knitted fabrics reflect the different roughness of fabrics. Table 1 and Figure 3a show that the relationship between the surface roughness of the fabrics when untreated and when treated with APP electric power of 1.0–4.0 Kw, with APP treatment time set at 15 s. Table 2 and Figure 3b show that the relationship between the surface roughness of the fabrics untreated and treated with APP electric time of 15–90 s when APP treated power is 1.0 Kw. These images clearly show that the surface roughness of fabric increases when APP treatment time and power are increased. The SEM photographs indicate that APP treatment can increase roughness of the fibers surface by etching it. Therefore, the increase of fibers and fabrics surface roughness causes the increase of the friction between fibers, which may impact the withdraw force between fibers and fiber hairiness.

#### 3.1.3. Hairiness Length and Withdraw Force

**Figure 4:**
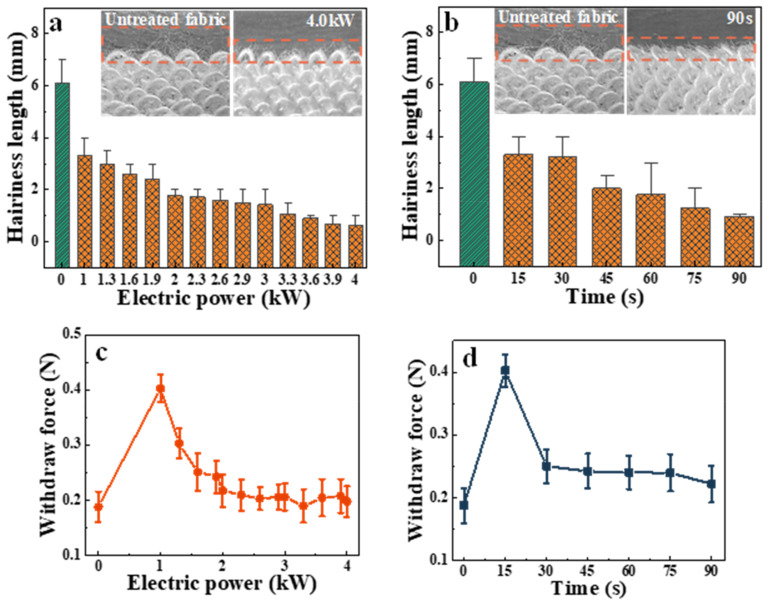
(**a**) Effect of APP treatment power on the hairiness length of fleece surface of fabrics; (**b**) effect of APP treatment time on the hairiness length of fleece surface of fabrics; (**c**) effect of APP treatment power on the withdraw force of the hairiness of fabrics; (**d**) effect of APP treatment time on the withdraw force of the hairiness of fabrics.

The hairiness length and the withdraw force on the fleece surface of the fabrics before and after APP treatment were also analyzed. The relationship between the hairiness length of fleece surface of fabrics before and after treatment with APP electric power of 1.0–4.0 kW is shown in Figure 4a. The relationship between the hairiness length of the fleece surface of fabrics and the treatment times after 15–90 s and without APP treatment is shown in Figure 4b. The relationship between the withdraw force of the hairiness of the fabrics before and after treatment with APP electric power of 1.0–4.0 kW is shown in Figure 4c. The relationship between the withdraw force of the hairiness of the fabrics and the treatment times after 15–90 s and without APP treatment is shown in Figure 4d. It can be observed that increasing the electric power and prolonging the treatment time of APP treatment will reduce the length of the hairiness of fleece surface of fabrics, and that the withdraw force of the fabrics decreased with the increase in line. The change in the values of withdraw force can be attributed to the different degrees of etching of fibers. The number of particle collisions on the fabric also increased when APP electric power was enhanced to improve the degree of fiber etching [37]. At higher power the fibers of fleece surface of fabrics will be broken or carbonized, resulting in the weakening of the withdraw force between the hairiness of fibers. Meanwhile, prolonging the APP treatment time will increase the number of collisions between the plasma and the fabrics surface [32], thereby weakening the fiber strength in the fabrics. Interestingly, the hairiness of fibers becomes shorter at higher power and longer times. The change in the hairiness of fibers is due to the breaking and carbonizing in APP atmospherics. Overall, increasing withdraw force and decreasing hairiness of fabrics can prevent hairfalling of the fibers.

### 3.2. Anti-Hairfalling Results

**Figure 5:**
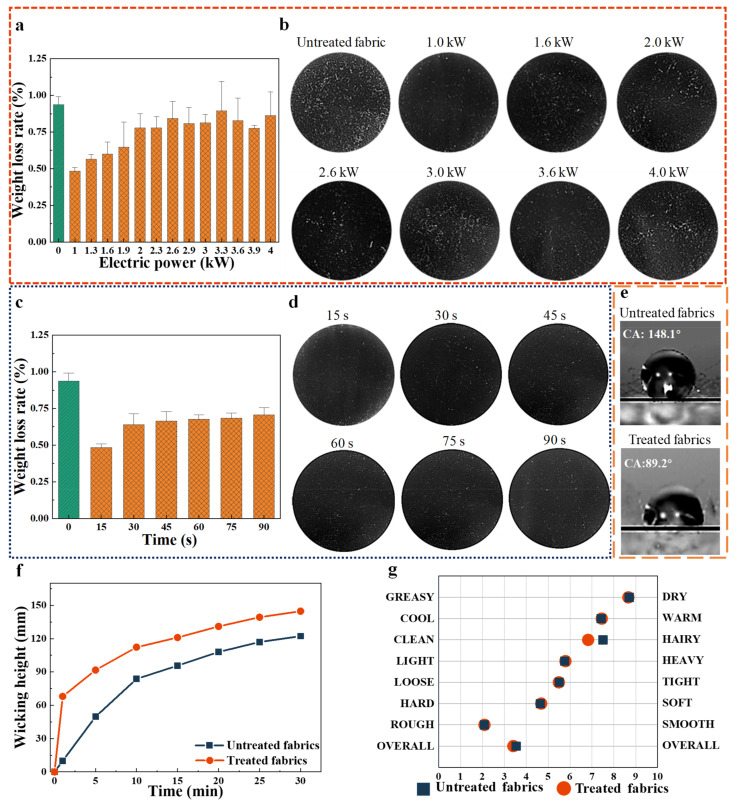
(**a**) Variation in weight loss rate of fabrics with APP treatment electric powers at 15 s; (**b**) different APP treatment electric powers to treat fabric loss hair condition at 15 s; (**c**) variation in weight loss rate of fabrics with APP treatment times at 1.0 kW; (**d**) different APP treatment time to treat fabric loss hair condition at 1.0 kW; (**e**) the contact angle of fabrics; (**f**) effect of wicking height of APP-treated and untreated fabrics; (**g**) effect of handle of untreated fabrics and APP-treated fabrics.

The changes in withdraw force and hairiness of fibers after the APP treatment indicated that the anti-hairfalling properties of polyester–cotton fleece knitted fabrics will be influenced by APP treatment parameters. Therefore, the effect of APP-treated power and time on the anti-hairfalling properties of the APP treated polyester–cotton fleece knitted fabrics was explored systematically. The weight loss rates and the optic images of fabrics treated with different APP treatment electric powers are shown in Figure 5a and Figure 5b, respectively. The lowest weight loss rate of the fabrics (0.48%) was recorded when the power was 1.0 kW. It is 48.4% lower compared with the untreated fabrics (with a loss rate of 0.93%). However, the weight loss rate of the fabrics tended to increases when the APP treatment power was increased. The change in the values of weight loss rate can be attributed to the different degrees of etching of fibers. The higher the energy of the active particles in the plasma atmosphere, the stronger the degree of fiber etching [37,38]. In addition, overdosed fiber etching can cause carbonization of the fibers and weakening of the withdraw force of the hairiness of the fabrics, leading to higher chance of hairfalling. Similarly, the weight loss rates and SEM images of fabrics treated with different APP treatment times are shown in Figure 5c and Figure 5d, respectively. It can be observed that increasing the treatment times with APP contributed to a relatively linear aggravation in weight loss rates of fabrics. The weight loss rate of the fabrics increases when the APP treatment time was exceeds 15 s. The bombardment of energetic species for a longer time causes breaking and carbonizing of fibers, resulting in the decrease of strength of the fibers reflected by the weakening of withdraw force of the hairiness of fiber and the increase in weight loss rate of fabrics. Meanwhile, plasma operated at high power and long reaction times has been reported to adversely affect the mechanical properties of the fabrics due to extensive surface etching [39]. Therefore, prolonging the time of APP treatment will weaken the anti-hairfalling properties of the fabrics. Overall, the anti-hairfalling properties of polyester–cotton fleece knitted fabrics treated under the conditions of APP treatment with electric power of 1.0 kW and treatment time of 15 s can be improved by 48.3% compared with the untreated fabrics.

### 3.3. The Property of the Fabrics after APP Treatment

#### 3.3.1. Chemical Characterization

**Figure 6:**
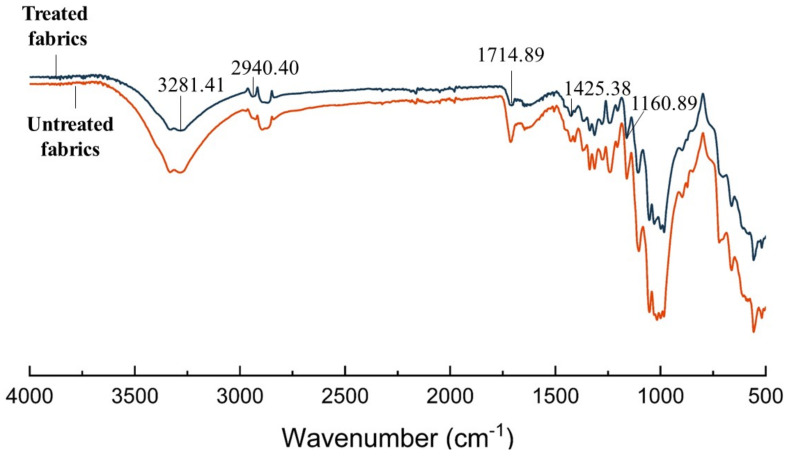
FTIR analysis of plasma treated fabrics and untreated fabrics.

The FTIR of untreated and plasma-treated fabrics are shown in Figure 6. It can be observed that there is no significant difference in the FTIR of the fabrics before and after plasma treatment. At 1714.89 cm^−1^, it is the telescopic vibration peak of C=O in the ester group, the telescopic vibration peak of C-H at 2940.40 cm^−1^. Comparing the infrared spectra of plasma treated and untreated fabrics, the characteristic peak intensity of C=O and C-H decreases, indicating that the C=O and C-H in the fabric were fractured after plasma treatment, which improved the surface properties and surface activity of the fabric fibers. Thus, plasma treatment can improve the hydrophilic properties of the fabrics to certain extent.

#### 3.3.2. The Effects of APP Activation on Wettability

The contact angle measurements are shown in Figure 5e. It can be observed that the contact angle (89.2°) of the polyester–cotton fleece knitted fabrics treated with APP treatment decreased by 39.7% when compared to the untreated polyester–cotton fleece knitted fabrics (the contact angle is 148.1°). The surface of the untreated polyester–cotton fleece knitted fabrics is hydrophobic. The reason is that cotton fibers contain more impurities, such as gum, grease, wax, pigments, oil stains, and minerals stained during spinning and weaving. These impurities seriously affect the wettability of cotton fibers, making the surface of untreated fabrics become hydrophobic. The decrease in the contact angle of treated fabric is caused by plasma etching. The etching will produce cracks on the fiber surface and increase the contact area between water droplets and fibers. Meanwhile, plasma treatment makes the pectin, wax, and other impurities on the surface of cotton fiber become oxidized and decomposed into gas and thus removed, and the etching effect is generated to make the surface of the fabric rough and so that the fiber pores increase, which is conducive to the full contact between water droplets and the fiber surface; In addition, the chemical bonds of the fiber surface molecules are opened and combined with free radicals such as oxygen and nitrogen in the plasma to form hydrophilic groups which is -OH on the fiber surface, thereby significantly improving the surface energy and the transportation ability of the fibers to water. Thus, the contact angle of the treated fabrics to water is decreased. The wicking height changes of water for untreated and treated fabrics are shown in Figure 5f. The wicking height increases linearly with the rising time for the untreated fabrics and reached 122.3 mm within 30 min. However, the wicking height increased rapidly to more than 121.0 mm at 15 min and then gradually increased with time for all APP treated fabrics. The wicking height at 15 min, 20 min, and 25 min APP treated fabrics are 121.0, 131.0, and 139.3 mm, respectively. Meanwhile, the wicking height of the APP treated fabrics are increased by 18.3% compared with untreated fabrics at 30 min. The reason why APP etching can form pits and cracks that could help in faster wetting of fabric is due to the capillary transport of water, and is sufficient for the maximum measurable improvement in wetting time [40]. It can be concluded from the contact angles and wicking heights that APP treatment enhances the wettability of fabrics. The increase in wettability of fabrics after the APP treatment can be also confirmed by SEM images and the roughness of fabrics.

#### 3.3.3. Handle

The value of seven indexes employed to evaluate the handle of samples and their overall ratings are presented in Figure 5g and Table 3. The values of indexes in Figure 5g show that treated fabrics have an overall score close to that of the untreated fabrics, indicating that APP treatment of fabrics has a limited effect on the handle of the fabrics. Notably, the Clean/Hairy index of treated fabrics shifted towards the threshold of Clean. The reason is that active and energetic species are generated at a higher glow discharge power in the plasma region, which results in effective physical bombardments or various chemical modifications on size fabrics and/or even on the fiber surfaces. In addition, the plasma physical and chemical etching effects resulted in the increased tendency of fabric weight loss through enhanced gas vaporization, ablation, and the effective carrying away of broken size particles and other impurities from the fabrics directly [6]. Therefore, the surface of treated fabrics is smoother and cleaner than untreated fabrics. This change in the smoothness of treated fabrics is reflected by the shifts of corresponding indexes towards the thresholds of Smooth and Clean in Figure 5g. Overall, plasma treatment can clean the surface of fabrics, and affects only the surface (up to several tens of nm) of the treated material while the bulk properties of the materials remain unchanged [41,42]. Thus, the overall handle of the treated fabrics is not distinctly affected.

#### 3.3.4. Tensile Property

The tensile test of the untreated and APP treated fabrics are shown in Table 4. The values of breaking elongation and breaking elongation index of APP-exposed polyester–cotton fleece knitted fabrics mentioned in the table clearly indicate the reduction in breaking elongation and breaking elongation index. The fabrics were exposed to APP for 15 s. The bombardment of energetic species causes etching at the surface of fibers that results in the decreases in breaking elongation and breaking elongation index value as compare to untreated fabrics. Generally, APP treatment of fabrics has a limited effect on the tensile properties of the fabrics. Thus, the APP treatment can functionalize the surface of the material while the original physical properties of the fabrics did not deteriorate much after the treatment.

## 4. Conclusions

The APP treatment significantly modifies the anti-hairfalling of polyester–cotton fleece knitted fabrics. The APP treatment can etch fiber which can form cracks and pits on the surface of fibers and decrease hairiness length of fabrics. The roughness formed at the surface of fiber and fabrics enhances withdraw force between the fibers and hairiness of fibers. Increasing withdraw force and decreasing hairiness length of fabrics can reduce hairfalling of the fibers. It has been observed that the hairfalling of the fabrics reduces substantially after being APP-treated. The anti-hairfalling properties of polyester–cotton fleece knitted fabrics are the best (the weight loss rate is 0.48%) when the anti-hairfalling property of the polyester–cotton fleece knitted fabrics is improved by 48.3% compared with the untreated fabrics (the weight loss rate is 0.93%) under the conditions of APP treatment with electric power of 1.0 kW and treatment time of 15 s. The contact angle is reduced by 39.7% and the wicking height is increased by 18.3% compared with the untreated fabrics under the same conditions. In addition, the handle and tensile properties of the APP-treated fabrics clearly showed that the fabrics of APP treatment have a limited effect on the handle of the polyester–cotton fleece knitted fabrics and physical properties. Therefore, the APP treatment is a feasible method for preventing hairfalling of polyester–cotton fleece knitted fabrics.

## Figures and Tables

**Table 1:** The roughness of fabrics under different APP-treated power.

Untreated Fabric	1.0 Kw	1.3 Kw	1.6 Kw	1.9 Kw	2.0 Kw	2.3 Kw	2.6 Kw	2.9 Kw	3.0 Kw	3.3 Kw	3.6 Kw	3.9 Kw	4.0 Kw
2.08	2.10	2.37	2.41	2.43	2.46	2.49	2.56	2.59	2.60	2.71	2.73	2.75	2.81

**Table 2:** The roughness of fabrics under different APP-treated time.

Untreated Fabric	15 s	30 s	45 s	60 s	75 s	90 s
2.08	2.10	2.22	2.49	2.52	2.61	2.74

**Table 3:** The handle index of the fabrics.

Handle Index	Grey Fabrics	Treated Fabrics
OVERALL	3.53	3.41
ROUGH/SMOOTH	2.08	2.10
HARD/SOFT	4.64	4.68
LOOSE/TIGHT	5.50	5.49
LIGHT/HEAVY	5.75	5.79
CLEAN/HAIRY	7.50	6.82
COOL/WARM	7.42	7.45
GREASY/DRY	8.70	8.66

**Table 4:** Tensile property of fabrics.

Fabric Type	Breaking Strength/ N	Breaking Elongation/ mm
Untreated fabrics	153	72.8
Treated fabrics	150	64.6

## Data Availability

The data presented in this study are available on request from the corresponding author.
